# Parasitism preference of Chalcid hymenopteran Dirhinus giffardii (Silvestri) confirms higher parasitism against housefly (Musca domestica) (Diptera: Muscidae) pupae

**DOI:** 10.1371/journal.pone.0262034

**Published:** 2022-01-20

**Authors:** Imran Rauf, Niaz Hussain Khuhro, Raza Muhammad Memon, Imtiaz Ahmed Khan

**Affiliations:** Plant Protection Division, Nuclear Institute of Agriculture (NIA), Tandojam, Pakistan; Government College University Faisalabad, PAKISTAN

## Abstract

The housefly, *Musca domestica* (Diptera: Muscidae), is capable of transmitting many pathogens that cause severe diseases in humans and animals. Mostly the management tactics rely on synthetic chemicals, but these chemicals creates lethal effects on biological ecosystem. For natural and safe options, bio-control strategy is one of the choice. The present study was a part of such effort to use this strategy and validate the biological performance of the potential pupal parasitoid *Dirhinus giffardii* (Silvestri) against house fly and provide alternative and safe control of filthy flies. This is the first report on parasitism potential and preference of *D*. *giffardii* against house fly. The *D*. *giffardii*, early reported as an effective pupal parasitoid of tephritid flies, here in the case presented, showed overall 70% reduction in the house fly population by parasitizing pupae. The parasitism efficiency and longevity of hymenopteran parasitoid was remarkably noted two-fold higher and one-fold more female production on house fly pupae as compared to primary hosts (Tephritids). Furthermore, sex ratio of the resultant progeny was also confirmed the dominancy of female by 74% as compared to males. Based on the novel findings we therefore conclude that *D*. *giffardii* is the best bio-control agent for controlling house flies.

## Introduction

In arthropods, class Insecta (order Diptera) is mainly related to disease transmission. Some members of families like Culicidae [1–3], Glossinidae [4], Psychodidae (subfamily: Phlebotominae) [5, 6], Calliphoridae [7, 8] and Muscidae [9–11] from order Diptera are capable to transmit pathogens either biologically or mechanically. The house fly *Musca domestica* L. (Diptera: Muscidae) are synanthropic flies responsible for the mechanical transmission of more than 100 pathogens [12–14]. They mainly feed on filth foods, animal and human wastes, decaying matters and garbage. Being a synanthropic and endophilic in nature, they love to survive and complete its whole life in or near human habitations [15]. They can fly long distances [16] and can be able to pick thousands of microorganisms with its mouthparts, legs, and other body organs (fine hairs on abdomen and thorax) [14]. Contamination of food and drinking water due to house fly keeps human life on risk of infections. Improper fly control measures creates recurrent episodes of epidemic such as cholera in the world. Several reports have been validated the involvement of house fly in spreading epidemics and outbreaks of certain human diseases [17–20]. It is estimated that, worldwide, millions of people regularly suffer and die annually with cholera, diarrhea, typhoid and bacterial dysentery, whereas, billions of dollars are being utilized to control these diseases. These diseases are mostly creating havoc situation in developing countries [21], where, poor sanitation and dense human population make environment conducive for house fly reproduction. Although chemical control exhibits quick results in acute cases but unfortunately these chemical possesses carcinogenic, mutagenic and teratogenic properties. On the other hand house fly is almost a permanent problem therefore use of such chemical will be deleterious special where infants resides. In this situation ecofriendly methods with excellent efficacy is needed. Bio-control is best natural answer to this problem. The generalist parasitoid *D*. *giffardii* is used as bio-control agent and target most of the flies belongs to order Diptera. It was initially reported as effective pupal parasitoid against a number of tephritid flies like Ceratitis *anonae*, *Bactroceracucurbitae*, *Ceratitis capitata*, *Bactrocera tryoni*, *Bactrocera dorsalis*, and *Bactrocera zonata* [22–27]. But still there is lack of evidence regarding parasitism efficiency, longevity, and performance on house fly pupae. There are some reports about the presence of *D*. *giffardii* on house fly pupae [28] but no enough data has been provided that confirmed the effectiveness of parasitoid. Keeping in mind healthy and clean environment concept, a study on chalcid hymenopteran, *D*. *giffardii* was conducted and its biological potential was analyzed on house fly. This bio-control agents was earlier reported as potent pupal parasitoid of (Tephritid) *C*. *capitata* [29] from West Africa (Nigeria), and now found to have shown exceptionally high parasitism potential against house fly pupae as compared to its parental host.

## Materials and methods

The study was conducted at “fruit fly and its parasitoid rearing laboratory” Nuclear Institute of Agriculture (NIA), Tandojam-Pakistan. The *D*. *giffardii* is a generalist parasitoid attacking puparia of cyclorrhaphous flies. An experiment was designed to evaluate the incidence, parasitism efficiency and host preference of *D*. *giffardii* on house fly pupae and correlate it with parent hosts (*B*. *zonata* and *B*. *cucurbitae*) pupae. The study was based on two experiments. In the first experiment, parasitism preference of *D*. *giffardii* was evaluated against three hosts, whereas, in the second experiment the parasitism efficiency of resulted progeny was tested.

### Fruit flies and parasitoid culture

The stock colony of fruit flies (*B*. *zonata* and *B*. *cucurbitae*) and its pupal parasitoid (*D*. *giffardii*) were obtained from the culture maintained at “Fruit fly rearing laboratory, NIA”. Both fruit fly species are being reared on artificial diet [30] and their culture is being used for mass rearing of pupal parasitoids.

### Housefly culture

Wheat bran based artificial diet [30] was used as egg laying substrate for houseflies. For egg laying, 250mg diet in (6″x 6″x 2″) tray was placed for two days in open area near garbage. After two days exposure to adult houseflies the same diet was then shifted to laboratory and kept under cover with muslin cloth at 28 ± 2°C and 70% relative humidity. After 2–3 days, tray with hatched eggs was shifted to larval rearing room at 23 ± 2°C and 70% relative humidity. When larval development was completed, tray with maggots was shifted to pupal collection room. Tray was placed on saw dust in plastic chamber (3´x 2´x 1´) for pupal collection. The pupae were sifted and separated carefully and used for experiments.

### Experiment no. 1

Thirty pairs of newly emerged parasitoids were collected from already maintained parasitoid culture and released in separate single cage (12″x12″x 12″). Two-day old, one hundred (100) pupae of each fly (house fly, *B*. *zonata* and *B*. *cucurbitae*) were offered as host and allowed for 3days as free choice for parasitism. After three days exposure, parasitized pupae were shifted to separate cages (12″x12″x 12″). After completion of pre-emergence development period (15 days), observations were taken on the basis of “emergence percentage” and “sex ratio” of emerged parasitoids up to ten consecutive days. The parasitism rate was calculated by using formula as [31].


Parasitismrate=numberofparasitoids(emerged+unemerged)/totalnumberofpupae×100

Whereas, percentage increase in “emergence percentage” and “sex ratio” was calculated by following formula:

%increase=Increase÷originalnumber×100

Before offering for parasitism, ten pupae (randomly selected) of each fly were weighed and their diameter (length and width) were measured with the help of Vernier caliper. Size of the parasitoids were measured in terms of body length (in mm) from the front of the head to the end of the abdomen. The experiment was conducted with five replicates at 26 ± 2°C with 60–65% relative humidity.

### Experiment no. 2

In the second experiment, single pair of newly emerged *D*. *giffardii* from each host (house fly, *B*. *zonata* and *B*. *cucurbitae* pupae) were selected from experiment no.1and further used to analyse their parasitism potential. Two-day old, ten pupae of *B*. *zonata* were offered to each pair of parasitoid on daily basis upto female life span and data was collected on the basis life parameters like life span of parasitoid, parasitism period, per day parasitism rate and total number of pupae parasitized in whole life. The experiment was conducted with five replicates at 26 ± 2°C with 60–65% relative humidity. Efficiency of parasitoids emerged from house fly pupae were compared with parasitoids emerged from *B*. *zonata* and *B*. *cucurbitae* pupae.

### Data analysis

Statistical comparisons were analyzed by using SPSS (Statistical for social sciences) version 23 and Statistix 8.1. Mean and standard deviation was calculated. *P*-values less than 0.05 were considered statistically as significant.

## Ethical approval

The authors would state that they only worked with small arthropods (insects) and the international guiding principles are not applicable for these organisms.

## Results

### Experiment no. 1

**Fig 1:**
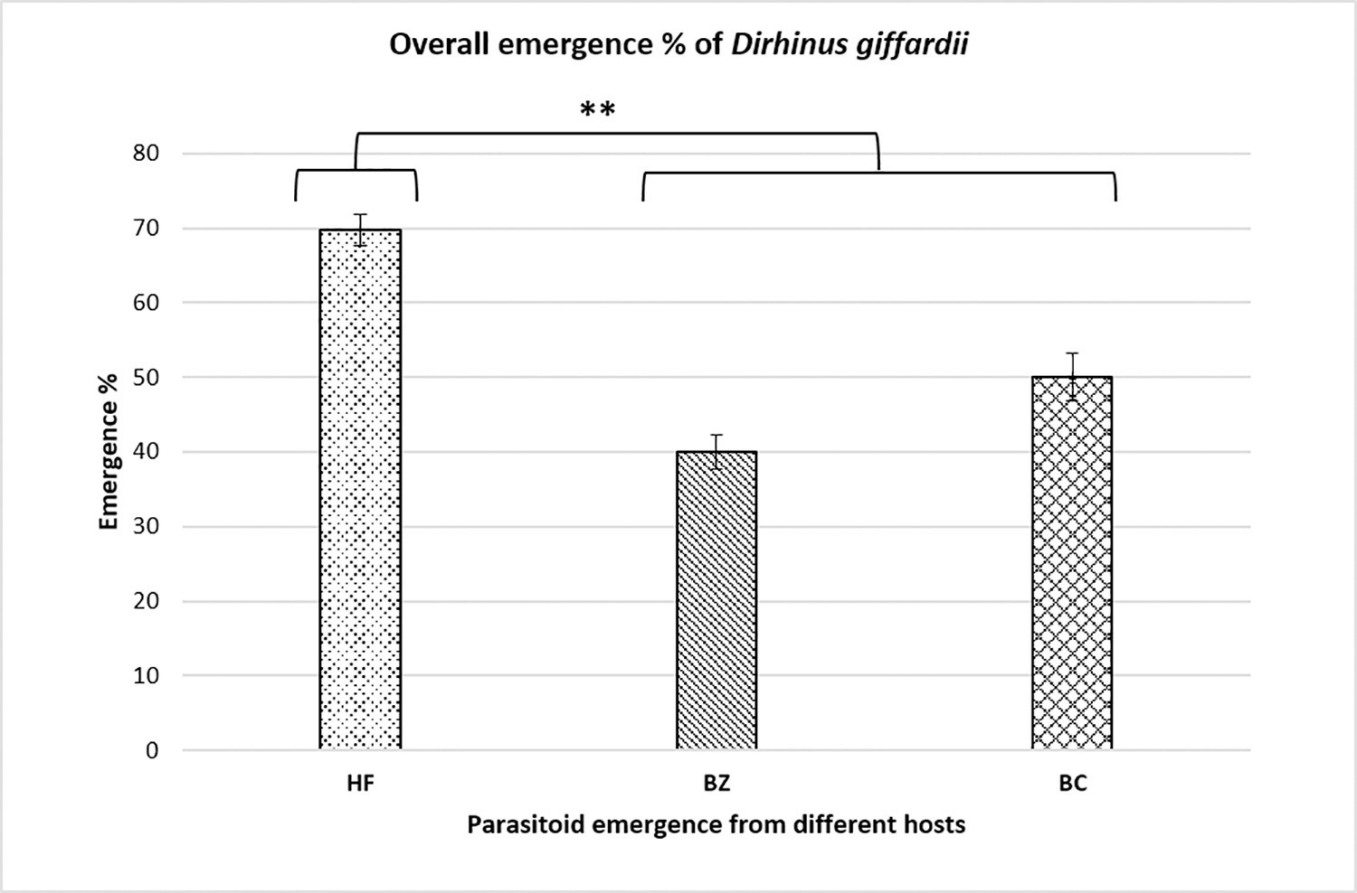
Overall parasitism percentage of *Dirhinus giffardii* on different hosts. HF = Housefly, BZ = *Bactrocera zonata* (Peach fruit fly) and BC = *Bactrocera cucurbitae* (Melon fruit fly). Mean ± S.E of N = 05. **P < 0.01, Mann-Whitney *U*-test.

**Fig 2:**
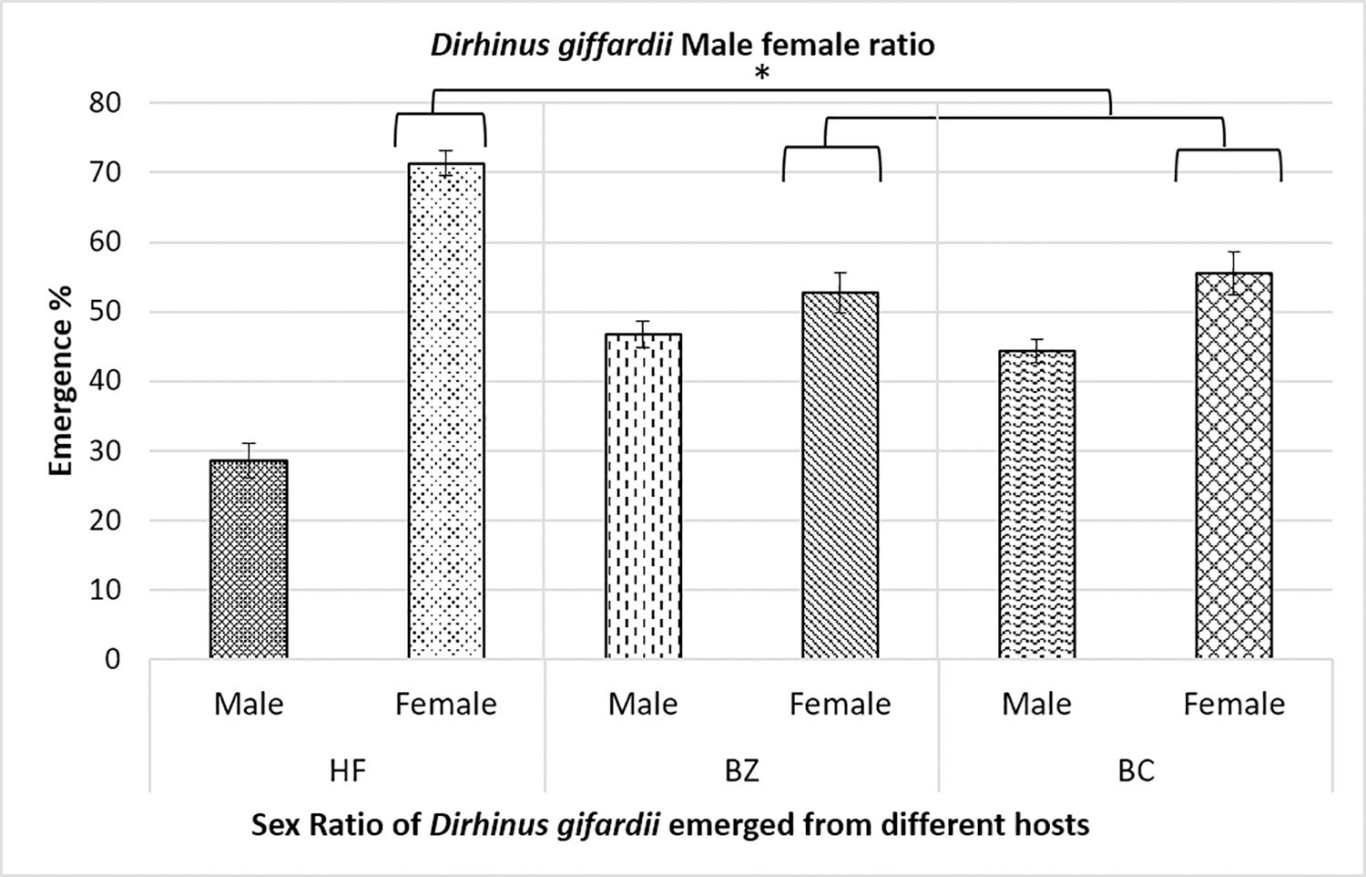
Male-Female ratio of *Dirhinus giffardii* emerged from different hosts. HF = Parasitoids emerged from Housefly, BZ = Parasitoids emerged from *Bactrocera zonata* (Peach fruit fly) and BC = Parasitoids emerged from *Bactrocera cucurbitae* (Melon fruit fly). Mean ± S.E of N = 10. *P < 0.05, Mann-Whitney *U*-test.

**Fig 3:**
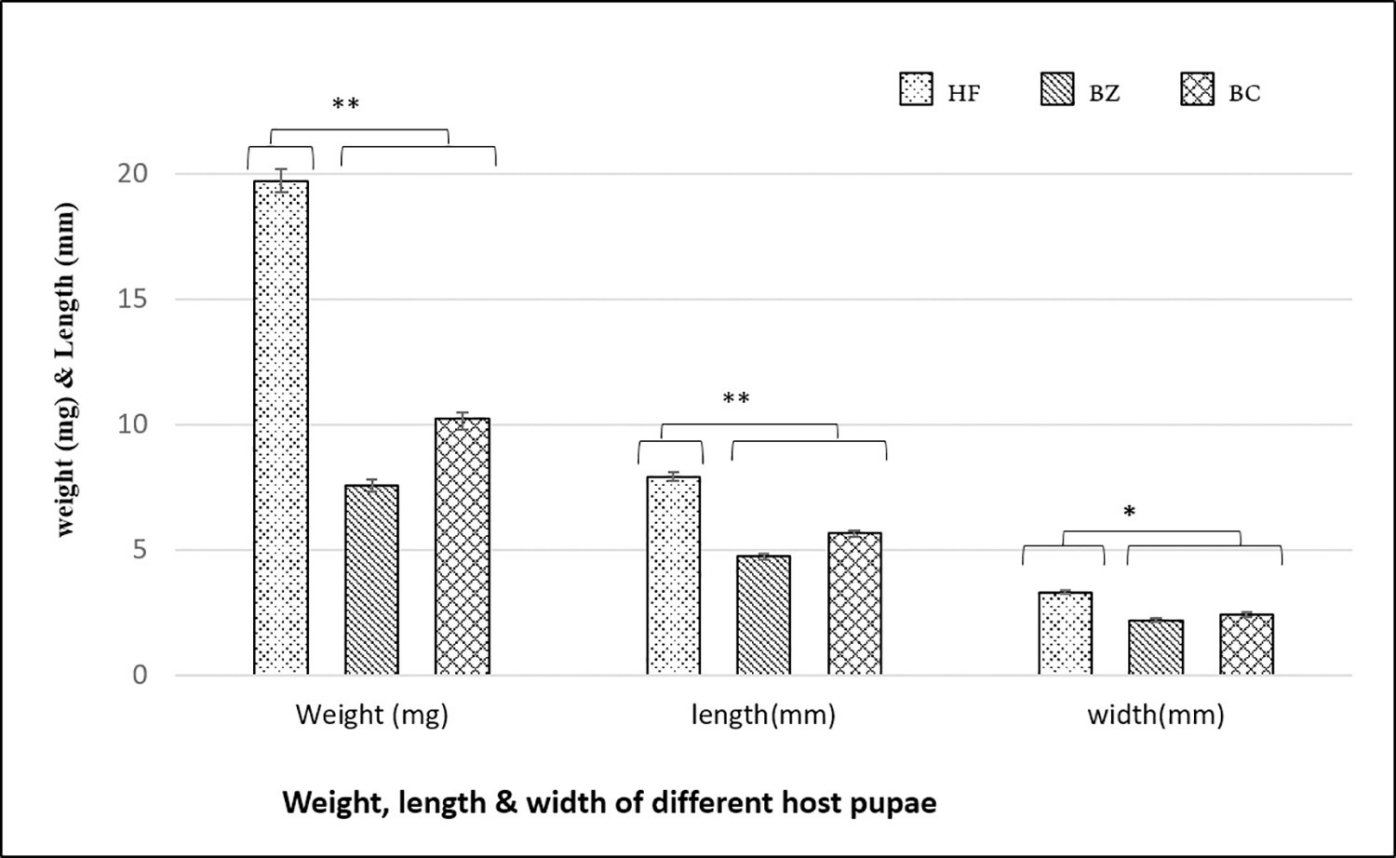
Weight, length and width of different host pupae. HF = Housefly pupa, BZ = *Bactrocera zonata* (Peach fruit fly) pupa and BC = *Bactrocera cucurbitae* (Melon fruit fly) pupa. Mean ± S.E of N = 10. **P < 0.01, *P < 0.05 Mann-Whitney *U*-test.

**Fig 4:**
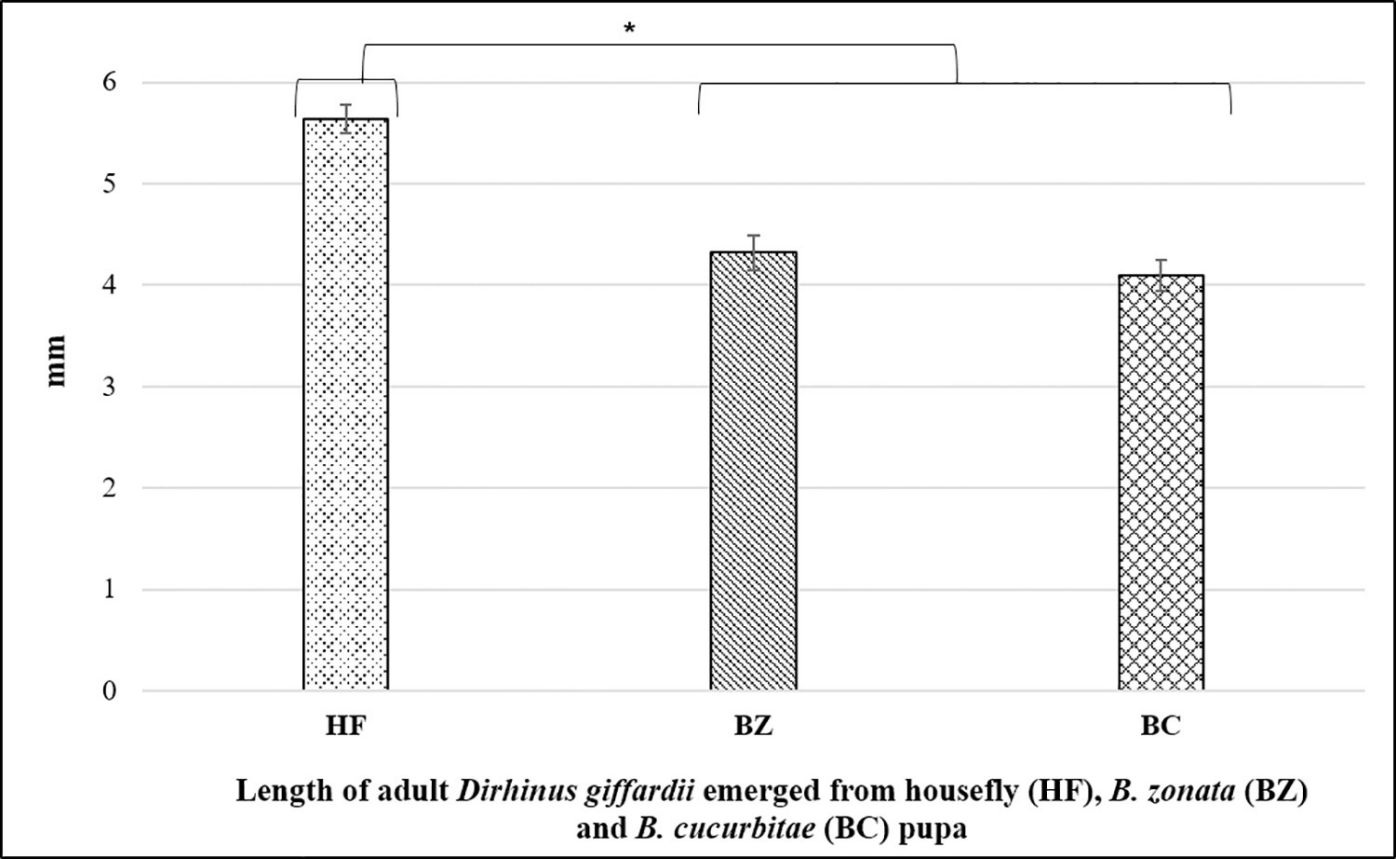
Length of adult parasitoids emerged from different hosts. HF = Housefly pupa, BZ = *Bactrocera zonata* (Peach fruit fly) pupa and BC = *Bactrocera cucurbitae* (Melon fruit fly) pupa. Mean ± S.E of N = 10. **P < 0.01, Mann-Whitney *U*-test.

During the first experiment of the study, parasitism preference and potential of *D*. *giffardii* were scrutinized against house fly pupae and was compared with primary hosts (*B*. *zonata* and *B*. *cucurbitae* pupae). According to the observations made, on an average, 70% of house fly pupae were parasitized by *D*. *giffardii* as compare to *B*. *cucurbitae* (50%) and *B*. *zonata* (40%) (Fig 1), whereas, female ratio in emerged parasitoids was one-fold higher (70%) in house fly pupae than that of *B*. *zonata* and *B*. *cucurbitae* (Fig 2). Overall, 75% more *D*. *giffardii* were preferably attracted towards house fly pupae as compared to its primary host *B*. *zonata* pupae as a free choice (Fig 1). The average weight and length of offered house fly, *B*. *zonata* and *B*. *cucurbitae* pupae were 20.20mg and 7.9mm, 8.17mg and 5.75mm, and 10.23mg and 5.95mm respectively (Fig 3). A remarkable large sized adult parasitoids (5.64mm) were emerged from house fly pupae as compare to parasitoids emerged from *B*. *zonata* (4.32mm) and *B*. *cucurbitae* pupae (4.0mm) (Fig 4).

### Experiment no. 2

**Fig 5:**
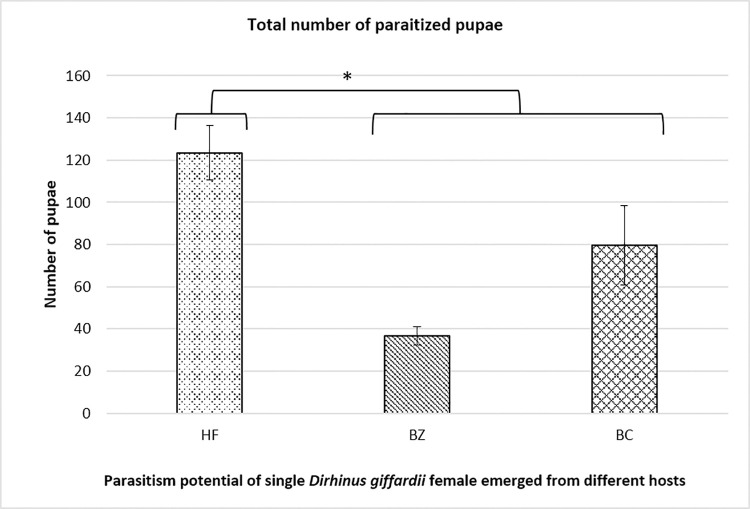
Number of pupae parasitized during whole life by single female *Dirhinus giffardii* emerged from different hosts. HF = Parasitoids emerged from Housefly, BZ = Parasitoids emerged from *Bactrocera zonata* (Peach fruit fly) and BC = Parasitoids emerged from *Bactrocera cucurbitae* (Melon fruit fly). Mean ± S.E of N = 05. *P < 0.05, Mann-Whitney *U*-test.

**Fig 6:**
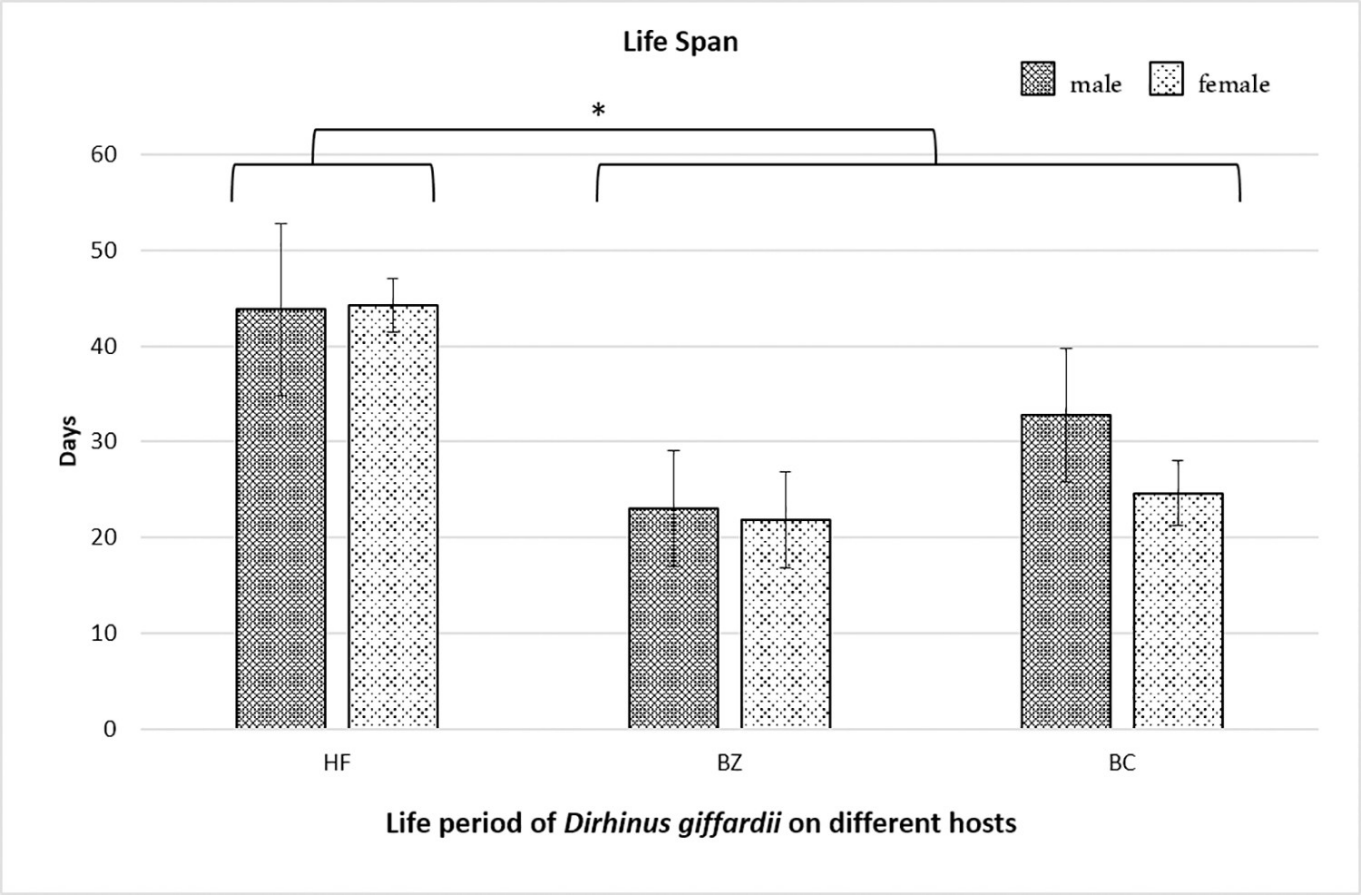
Life span of male and females *Dirhinus giffardii* emerged from different hosts. HF = Parasitoids emerged from Housefly, BZ = Parasitoids emerged from *Bactrocera zonata* (Peach fruit fly) and BC = Parasitoids emerged from *Bactrocera cucurbitae* (Melon fruit fly). Mean ± S.E of N = 05. *P < 0.05, Mann-Whitney *U*-test.

**Fig 7:**
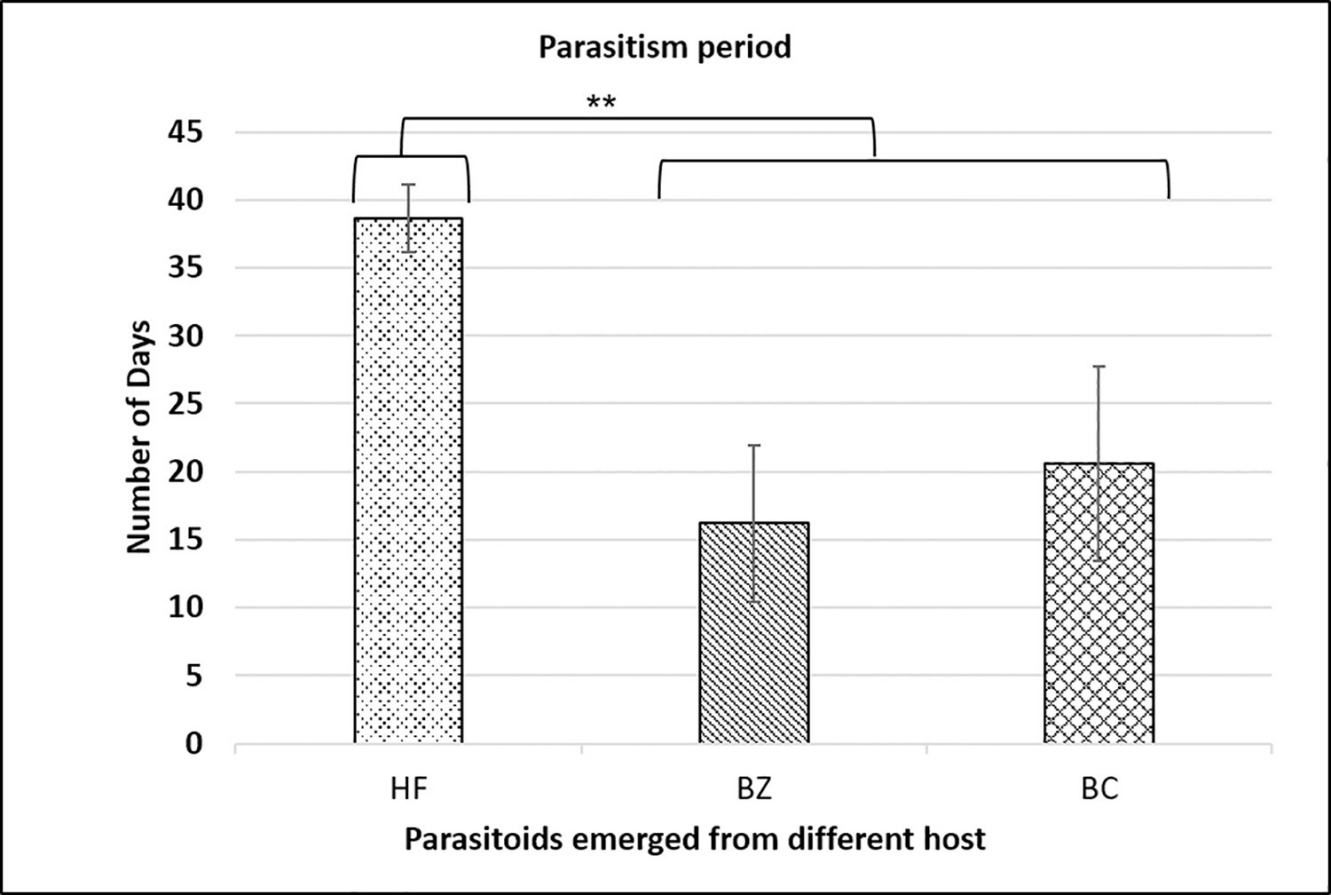
Parasitism period of *Dirhinus giffardii* female emerged from different hosts. HF = Parasitoids emerged from Housefly, BZ = Parasitoids emerged from *Bactrocera zonata* (Peach fruit fly) and BC = Parasitoids emerged from *Bactrocera cucurbitae* (Melon fruit fly). Mean ± S.E of N = 05.

**Fig 8:**
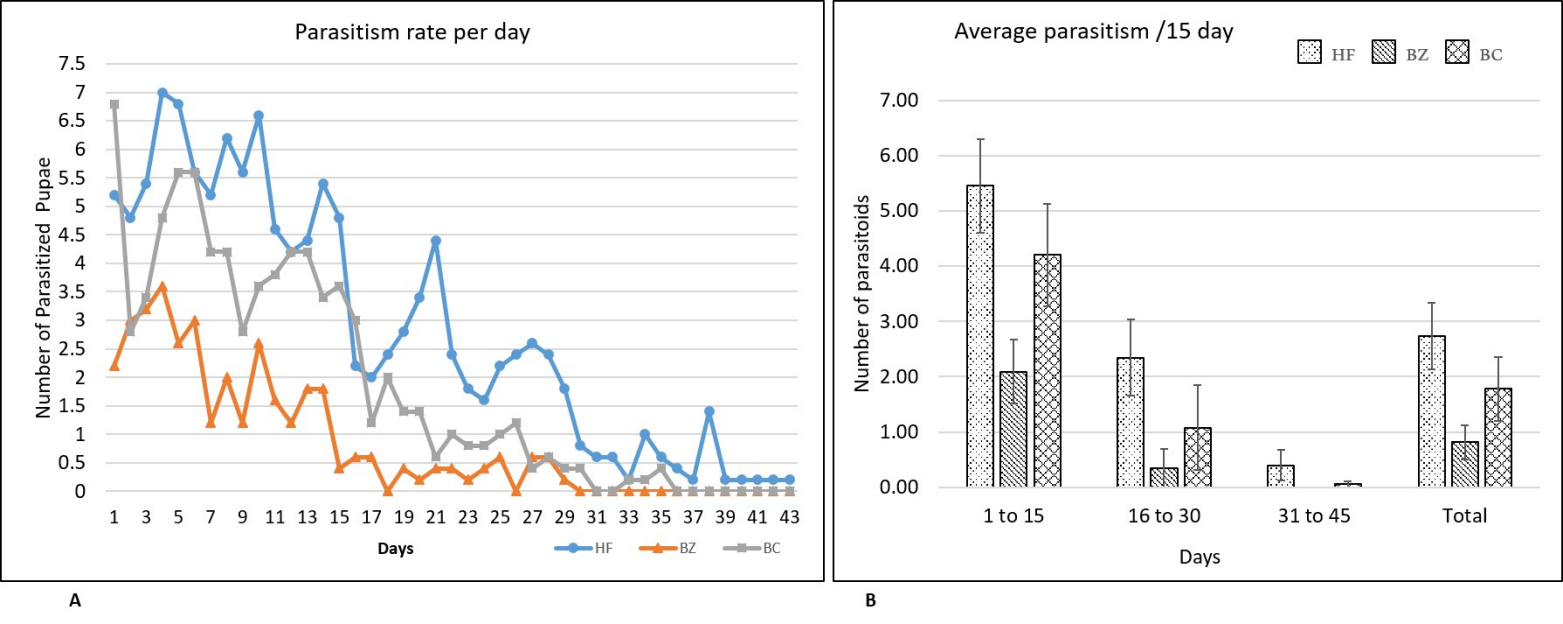
Parasitism rate of single female *Dirhinus giffardii* emerged from different hosts. A) Total number of parasitized pupae per day B). Average number of parasitized pupae per fifteen days. HF = Parasitoids emerged from Housefly, BZ = Parasitoids emerged from *Bactrocera zonata* (Peach fruit fly) and BC = Parasitoids emerged from *Bactrocera cucurbitae* (Melon fruit fly). Mean ± S.E of N = 05.

In the second experiment, the biological performance of the newly emerged *D*. *giffardii* couple from house fly pupae was further investigated and compared with parasitoids emerged from other parent hosts (*B*. *zonata* and *B*. *cucurbitae*). According to the data recorded, during the whole life of female parasitoid, the fecundity of a single female parasitoid emerged from house fly was two-fold higher as compared to female parasitoid from *B*. *zonata* pupae, and, one-fold increased than that of from *B*. *cucurbitae* pupae (Fig 5). The lifespan of male and female parasitoids emerged from house fly were same (44 days), but was significantly higher than that of parasitoids emerged from *B*. *zonata* (23 days for male & 21.8 days for females) and *B*. *cucurbitae* (32.8, 24.6 for male and female respectively) (Fig 6). In case of reproductive period of parasitoids, female emerged from house fly pupae had the longest reproductive period (38.6 days) followed by *B*. *cucurbitae* (20.6 days) and *B*. *zonata* (16.2 days) respectively (Fig 7). Female parasitoid emerged from house fly pupae shown significantly higher fertility rate (parasitized 5.45 pupae /day) in first fifteen days of life as compare to female emerged from *B*. *zonata* (2.09 pupae/day) and *B*. *cucurbitae* (4.20 pupae/day) (Fig 8A). The fertility was gradually decreases with passage of time. Overall, female parasitoid emerged from house fly exhibit one-fold higher fertility and have longer reproductive phase than that of female parasitoids emerged from other hosts (Fig 8A & 8B).

## Discussion

Parasitoids have an ability to discriminate between different hosts. Host searching ability of parasitoids are mainly focused on physiological and nutritional interrelationship [32]. The results presented here are clearly reveal that the *D*. *giffardii* proved itself to be more lethal against house fly as compared to its primary host *B*. *zonata* and *B*. *cucurbitae*. Early reports still validate that this parasitoid act as a potential bio-control agent of tephritid flies, but here, this is the first time we report parasitism potential of *D*. *giffardii* against House fly. The selection of suitable host by generalist parasitoids are influenced by multiple factors like host size, host density, host nutrition, host immunoreactions, host specie, host development stages and environmental elements etc. [33]. Based on the parameters that we are evaluated in this study, it is evident that host size was the determining factor in attraction and preference of parasitoids for oviposition. One-fold reduction in house fly population than primary hosts may due to attraction of parasitoids towards larger host. The house fly pupae are comparatively larger in size than that of tephritid flies pupae (*B*. *zonata* & *B*. *cucurbitae*).

Larger sized pupae (hosts) not only favored attraction of female parasitoids but also positively influenced on further biological performance (sex ratio, life span, parasitism period, adult size, and parasitism potential per day) of the resultant progeny. According to our results, the overall performance of resulted progeny emerged from house fly pupae was also remarkably superior to that of primary hosts (*B*. *zonata* & *B*. *cucurbitae*). Sex ratio play an important role in parasitism performance. In the results presented here, house fly parasitism produced more female parasitoid population than that of primary hosts. The more parasitism and higher female ratio of *D*. *giffardii* on house fly hosts indicate that it is a good quality host. Female parasitoids have an ability to select whether to fertilize an egg or not, and this selection is purely based on host quality [34]. Female parasitoids prefer to produce female offsprings on nutrient-rich hosts since larger females can produce more eggs [34]. Same results were also supported by Olga [35] and Ueno [36] which confirmed that host body size influenced on female parasitoids fecundity and sex ratio, and those females which emerged from medium or larger hosts become more fecund. The same findings are also in favor that female parasitoids (mother) should lay primarily daughters (females) in larger hosts and sons (male) in smaller hosts, and the resultant females conferred more reproductive success than that of males [37–39].

Increase in parasitoid longevity emerged from house fly pupae also showed a positive correlation between host size and parasitoid longevity. As per findings of Shangkun et. al, [40] and Sagarra et. al [41], more offspring were produced by large size female parasitoid as compare to small size. Our results also validate Shangkun et. al findings that the adult parasitoids emerged from house fly pupae were bigger in size than that of emerged from *B*. *zonata* and *B*. *cucurbitae* and, the resultant bigger sized females parasitized more pupae as compare to smaller size females. Same as other findings, the life span of parasitoid progeny was also positive correlate with host size. The life span of resultant parasitoid progeny emerged from house fly pupae were proved to be significantly more extended than that of other hosts. The same observations were also documented by AbdelRehman [42] and King [37]. *D*. *giffardii* has also been reported on Black soldier fly which confirmed that this parasitoid have an ability to disturb life of other flies instead of tephritids [43]. Other species of genus “*Dirhinus*” such as *D*. *himalayanus* also proved to be an effective against HF [44, 45] which support our results that genus “Dirhinus” is effectively parasitize HF pupae.

## Conclusion

Cosmopolitan nature and feeding habit on filty material makes house fly a potent vector with wide range of diseases transmission ability. Bulk of documents are available on chemical control of house fly but adverse effects of these chemicals are still questionable. Biological control is the best alternative that provides natural and safe control when use alone or in combination. Pupal parasitoids are the most common bio-control agents used for the filth fly management and their use is increasing day by day. Although some pupal parasitoids are commercially available and marketed, but the *D*. *giffardii* was still not yet documented against house fly parasitoid. The present preliminary study exhibit the biological control potential of *D*. *giffardii* against house fly and validated some remarkable biological activities like 70% higher parasitism and 100% more female progeny production as compare to parental hosts. These novel findings promotes and confirmed *D*. *giffardii* itself as a better venator of house fly, whereas, its mass rearing and release could be the ecofriendly way to manage flies without compromising environmental and food safety as well as the human and animal health.
